# Lactation has persistent effects on a mother’s metabolism and mitochondrial function

**DOI:** 10.1038/s41598-017-17418-7

**Published:** 2017-12-07

**Authors:** Hayden W. Hyatt, Yufeng Zhang, Wendy R. Hood, Andreas N. Kavazis

**Affiliations:** 1 School of Kinesiology, Auburn University, Auburn, AL USA; 2 Department of Biological Sciences, Auburn University, Auburn, AL USA

## Abstract

Human epidemiological data show that breastfeeding reduces the prevalence of numerous diseases compared to mothers that give birth but do not participate in lactation. The goal of this study was to determine if differences in metabolism, mitochondrial function, and oxidative stress underlie the protective phenotype found in lactating women. Ten-week old female Sprague-Dawley rats were divided into three groups (n = 8 per group): 1) rats that did not reproduce (NR), 2) rats that were allowed to mate and become pregnant but did not suckle their pups after giving birth (NL), and 3) rats that were allowed to mate and become pregnant and suckled their pups for 21 days before weaning (L). All animals were sacrificed at approximately 7 months of age, a time corresponding to 15 weeks after the NL and L females gave birth. Liver mitochondrial respiration was higher in L rats when using NADH-linked substrates and these rats had lower serum glucose concentration. Additionally, the L group exhibited changes in liver, skeletal muscle, and white adipose tissue PPARδ protein levels that may, in part, explain the observed lower serum glucose concentration. These novel animal findings provide evidence of differences in metabolic processes that persist months after weaning.

## Introduction

Lactation imposes one of the most metabolically challenging events in a female’s lifetime. Importantly, human epidemiological data have demonstrated an association between breastfeeding and a lower risk of obesity, type II diabetes, hypertension, and several types of cancer^1–4^. While a plethora of data exists on how metabolic perturbations such as exercise, fasting, and calorie restriction can promote a healthy phenotype^5–8^, very little research has been conducted on the mechanisms that underlie the changes that occur with breastfeeding.

During pregnancy, a female’s body undergoes many dramatic changes in metabolism that may predispose her to certain health disparities, if not reversed. With the onset of pregnancy, maternal insulin secretion increases in order to support glucose and amino acid transport to the fetus. However, a combination of increased insulin secretion, increased circulating lipids, and consequently, increased visceral adiposity often leads to insulin resistance of maternal cells^9,10^. When breastfeeding is initiated, a metabolic shift occurs that alters resource allocation from storage to milk synthesis. Indeed, breastfeeding results in improved glucose handling via a decrease in insulin production, improved insulin sensitivity, and a drop in β-cell proliferation. In addition, lipid metabolism is decreased in metabolically active tissues and lipid stores are mobilized to facilitate lipid transport to the mammary gland for milk synthesis^11,12^. Thus, lactation aides in reducing postpartum adiposity; potentially reducing the risk of obesity^13^. Given this evidence, Stuebe and Rich-Edwards proposed the reset hypothesis which posits that lactation is a critical event in resetting metabolic processes that occur during pregnancy and reduce the prevalence of metabolic disease^14^.

Liver, skeletal muscle, and white adipose tissue (WAT) contribute more than 50% of the metabolic rate in mammals^15^, and thus modulation of the metabolic requirements of these tissues is important during the increased energy demands imposed by lactation. Considering that dysregulation of mitochondrial function and metabolism have been demonstrated to play implicit roles in health disparities (e.g., obesity and type II diabetes)^16,17^, these would appear to be important markers for understanding how lactation may protect against disease. Furthermore, conditions of oxidative stress have also been implicated in the development of these health disparities^18,19^, and thus may underpin a mechanism in which lactation may also play a role in reducing prevalence of disease.

Variation in metabolism, mitochondrial function, and oxidative stress between females that breastfed and those that did not breastfeed after giving birth have not been previously documented in detail. Thus, the goal of this investigation was to delineate long-term differences in markers of metabolism, mitochondrial function, and oxidative stress between females that gave birth and suckled their young, females that gave and did not suckle their young, and non-reproductive control females. If lactation acts to reset a female’s metabolism^14^, lactation should bring females back to the metabolic condition of non-reproductive females. Thus, we anticipate that markers of metabolism, mitochondrial function, and oxidative stress will be similar for rats that suckled their young and female rats that did not reproduce. Rats that gave birth but didn’t suckle their young are expected to display a phenotype indicative of current or future metabolic disease.

## Methods

### Animal husbandry

All experimental procedures were approved by Auburn University’s Institutional Animal Care and Use Committee and were carried under the guidelines of the American Physiological Society and the National Institutes of Health Guide for the Care and Use of Laboratory Animals. Ten-week old Sprague-Dawley rats were obtained from Envigo. Animals (n = 8 per group) were acclimated with their diet and the facility ten days prior to experimental start. Rats were housed under standard laboratory conditions (46 × 25 × 20 cm boxes, 12 L:12D cycle, 22 °C, 50% RH), and given *ad libitum* access to food (Teklad Global Diet 2018) and water. Animals were randomly assigned to one of three treatment groups: 1) rats that did not reproduce (NR), 2) rats that were allowed to mate and become pregnant but did not suckle their pups after giving birth (NL), and 3) rats that were allowed to mate and become pregnant and suckled their pups after giving birth for 21 days (L). Female rats were housed in a box with another female from the same group, but were separated for pregnancy and lactation to prevent cross fostering. The NL animals had their pups removed within 12 hours of birth, and the NL mothers were then re-paired with another NL female 2 days after parturition and no noticeable behavioral changes were observed. The L animals had their litter size adjusted to 8 on the day of parturition. All animals were age-matched and sacrificed at a time that corresponded to 12 weeks following 21 days of lactation in L animals. As a result, females in all groups were sacrificed when they were approximately 7 months old (NR = 205 ± 1 days, NL = 210 ± 4 days, L = 208 ± 3 days, p > 0.05).

### Whole animal metabolism

Whole-animal metabolic measurements were completed in a Sable System Promethion Metabolic Screening System (Las Vegas, NV, USA) housed in Auburn University’s Lab Animal Health Facility. Each rodent box was equipped with a flow-through respirometry system for continuously monitoring oxygen intake, carbon dioxide output, and an infrared grid that continuously monitored the animals’ activity^20^. This system provided data to monitor energy expenditure and respiratory quotient of each rat.

### Blood collection and analysis

Rats were fasted four hours (from 8:00 to 12:00) prior to blood collection. Animals were anesthetized using isoflurane vapors and body mass was quickly recorded. The anesthetized animals were than decapitated, and blood was collected, allowed to clot on ice, and then centrifuged. Following centrifugation the serum was frozen at −80 °C for subsequent analyses. Serum glucose (STA-680, Cell Biolabs, San Diego, CA, USA), serum insulin (EZRMI-13K, EMD Millipore, St. Charles, MO, USA), serum non-esterified fatty acids (NEFA) (STA-618, Cell Biolabs) were quantified using the manufacturer’s specifications.

### Tissue collection and handling

After the decapitation, the following tissues were excised and weighed: liver, triceps surae (‘calf’ muscle), retroperitoneal white adipose tissue (RetroP WAT) and perirenal white adipose tissue (PR WAT) pads. After the mass of each tissue was recorded, a sample from calf skeletal muscle and liver was used for mitochondrial isolation and the remainder of tissues were frozen in liquid nitrogen and stored at −80 °C for subsequent analyses.

### Mitochondrial isolation

Mitochondrial isolations for muscle were performed as previously described^21^. Excised muscles (~750 mg) were trimmed to remove fat and connective tissues, weighed, and placed in 10 volumes of solution I (100 mM KCl, 40 mM Tris HCl, 10 mM Tris base, 1 mM MgSO_4_, 0.1 mM EDTA, 0.2 mM ATP, and 2% (wt/vol) free fatty acid bovine serum albumin (BSA), pH 7.40). Muscles were minced with scissors and the mince was homogenized for 15 seconds with a polytron. Protease (Trypsin) was added (5 mg/g wet muscle), and the digested mince was mixed continually for 7 minutes. Digestion was terminated by the addition of an equal volume of solution I. The homogenate was centrifuged at 500 *g* for 10 minutes at 4 °C and the supernatant was rapidly decanted through a double layer of cheesecloth and centrifuged at 3,500 *g* for 10 minutes. The supernatant was discarded and the mitochondrial pellet was resuspended in solution I. The suspension was centrifuged at 3,500 *g* for 10 minutes. The supernatant was again discarded, and the pellet was resuspended in 10 volumes of solution II (similar to solution I, but without BSA). This resuspended pellet was subsequently centrifuged at 3,500 *g* for 10 minutes. The final mitochondrial pellet was suspended in 250 μl of a solution containing 220 mM mannitol, 70 mM sucrose, 10 mM Tris HCl, and 1 mM EGTA, pH 7.40. Mitochondria from liver were isolated as previously described^22^. Briefly, liver (~750 mg) was weighed and placed in 10 volumes of solution III (250 mM sucrose, 5 mM HEPES, and 1 mM EGTA), minced with scissors and the mince was homogenized with a Potter-Elvehjem PTFE pestle and glass tube (2 passes). The homogenate was centrifuged at 500 g for 10 minutes at 4 °C. The supernatant was rapidly decanted through a double layer of cheesecloth and centrifuged at 3,500 g for 10 minutes. The supernatant was discarded and the mitochondrial pellet was resuspended in solution III. The suspension was centrifuged at 3,500 g for 10 minutes. The final mitochondrial pellet was suspended in 250 μl of a solution containing 220 mM mannitol, 70 mM sucrose, 10 mM Tris HCl, and 1 mM EGTA, pH 7.40.

### Isolated mitochondrial oxidative phosphorylation

Mitochondrial oxygen consumption was measured as described by Messer *et al*.^23^. Briefly, mitochondrial oxygen consumption was measured polarographically in a respiration chamber (Hansatech Instruments, United Kingdom). Isolated mitochondria (20 µL) were incubated with 1 ml of respiration buffer adapted from Wanders *et al*.^24^ (100 mM KCL, 50 mM MOPS, 10 mM KH_2_PO_4_, 20 mM glucose, 10 mM MgCl_2_, 1 mM EGTA, and 0.2% fatty acid free BSA; pH = 7.0) at 37 °C in a respiratory chamber with continuous stirring. For state 3 respiration, 2 mM pyruvate and 2 mM malate (complex I substrates) or 5 mM succinate (complex II substrate) was used in the presence of 0.25 mM ADP, and state 4 respiration was recorded following the phosphorylation of ADP as described by Estabrook^25^. Respiratory control ratio (RCR) was calculated as state 3/state 4 oxygen consumption. Respiration values were expressed as a ratio to citrate synthase to compensate for mitochondrial enrichment in the samples.

### Mitochondrial oxidant emission

Oxidant emission by mitochondria was determined using the oxidation of the fluorogenic indicator Amplex Red (Molecular Probes, Eugene, OR) in the presence of horseradish peroxidase^26^. The assay was performed at 37 °C in 96-well plates using succinate as the substrate. Specifically, this assay was developed based on the concept that horseradish peroxidase catalyzes the hydrogen peroxide-dependent oxidation of nonfluorescent Amplex Red to fluorescent Resorufin Red. Resorufin Red formation was monitored at an excitation wavelength of 545 nm and an emission wavelength of 590 nm using a multiwell plate reader fluorometer (Synergy H1, BioTek, Winooski, VT, USA). We recorded the level of Resorufin Red formation, and hydrogen peroxide production was calculated with a standard curve.

### Enzymatic assays for electron transport chain complex activity

Complex I (NADH dehydrogenase) enzyme activity (EC 1.6.5.3) was measured as a function of the decrease in absorbance from NADH oxidation by decylubiquinone before and after rotenone addition^27^. Complex II (succinate dehydrogenase) activity (EC 1.3.5.1) was measured as a function of the decrease in absorbance from 2,6-dichloroindophenol reduction^27^. Complex III (ubiquinol cytochrome *c* oxidoreductase) activity (EC 1.10.2.2) was determined as a function of the increase in absorbance from cytochrome *c* reduction^27^. Complex IV (cytochrome *c* oxidoreductase) activity was determined as a function of the decrease in absorbance from cytochrome *c* oxidation^27^. Specificity of complex IV activity was determined by monitoring changes in absorbance in the presence of KCN^27^. Citrate synthase (EC 4.1.3.7) was measured as a function of the increase in absorbance from 5,5′-dithiobis-2-nitrobenzoic acid reduction^27^. Enzyme activities were expressed as a ratio to citrate synthase to compensate for mitochondrial enrichment in the cell samples.

### Protein abundance

The relative concentration of proteins was quantified by Western blot analysis^26^. To accomplish this, tissue was homogenized 1:10 (wt/vol) in 5 mM Tris HCl (pH 7.5) and 5 mM EDTA (pH 8.0), and protease inhibitor cocktail (14224–396, VWR, Radnor, PA, USA) and was centrifuged at 1500 *g* for 10 minutes at 4 °C. Protein content of the supernatant was quantified by the method of Bradford^28^. Proteins were separated by polyacrylamide gel electrophoresis via 4–20% polyacrylamide gels (BioRad, Hercules, CA, USA). After electrophoresis, the proteins were transferred to PVDF membranes. Non-specific sites were blocked in phosphate-buffered saline (PBS) solution containing 0.1% Tween 20 and 5% non-fat milk. Membranes were then incubated overnight at 4 °C with primary antibodies purchased from GeneTex (Irvine, CA, USA) directed against peroxisome proliferator activated receptor alpha (PPARα, GTX101096, 1:1000), peroxisome proliferator activated receptor delta (PPARδ, GTX113250, 1:2000), peroxisome proliferator activated receptor gamma, coactivator 1 alpha (PGC-1α, GTX37356, 1:1000), superoxide dismutase 1 (SOD1, GTX100554 1:2000), superoxide dismutase 2 (SOD2, GTX116093, 1:2000), catalase (CAT, GTX110704, 1:2000), and glutathione peroxidase (GPX, GTX116040, 1:2000). Following incubation with primary antibodies, membranes were washed (five minutes × 3) with PBS-Tween and then incubated with secondary antibodies for one hour in room temperature. After washing (five minutes × 3), a chemiluminescent system was used to detect labeled proteins (GE Healthcare, Buckinghamshire, UK). Images of the membranes were captured and analyzed by using the ChemiDoc-It2 Imaging System (UVP, LLC, Upland, CA). Protein expression was normalized to Ponceau staining and variability between membranes was normalized by using an internal control run with each membrane.

### Assessment of indices of oxidative damage

To determine the relative amount oxidative damage, we measured protein oxidation and lipid peroxidation. Lipid peroxidation was assessed by determining 4-hydroxynoneal (4-HNE; *trans*-4-hydroxy-2-nonenal, C_9_H_16_O_2_) expression via western blotting. Primary antibody for 4-HNE was purchased from Abcam (ab46545; 1:1000 dilution, Cambridge, MA, USA). Protein oxidation was measured by comparing relative expression of protein carbonyls using a commercially available kit (Oxy-Blot protein oxidation detection kit; Intergen, Purchase, NY, USA) via western blotting as described by the manufacturer’s instructions.

### Statistics

Comparison between groups for each dependent variable were made by one-way analysis of variance (ANOVA), with a Tukey post hoc test being used to determine differences between groups. However, in the case of state 4 succinate muscle, CAT liver, and 4-HNE muscle the Brown-Forsythe test was significant, indicating unequal variance. Thus, Kruskal-Wallis test was performed followed by the Dunn’s for multiple comparisons post-hoc. Respiratory quotient and energy expenditure data were analyzed by a mixed model ANOVA. Data are presented as means ± SD, and significance was established at p < 0.05.

## Results

### Body and tissue mass

**Figure 1:**
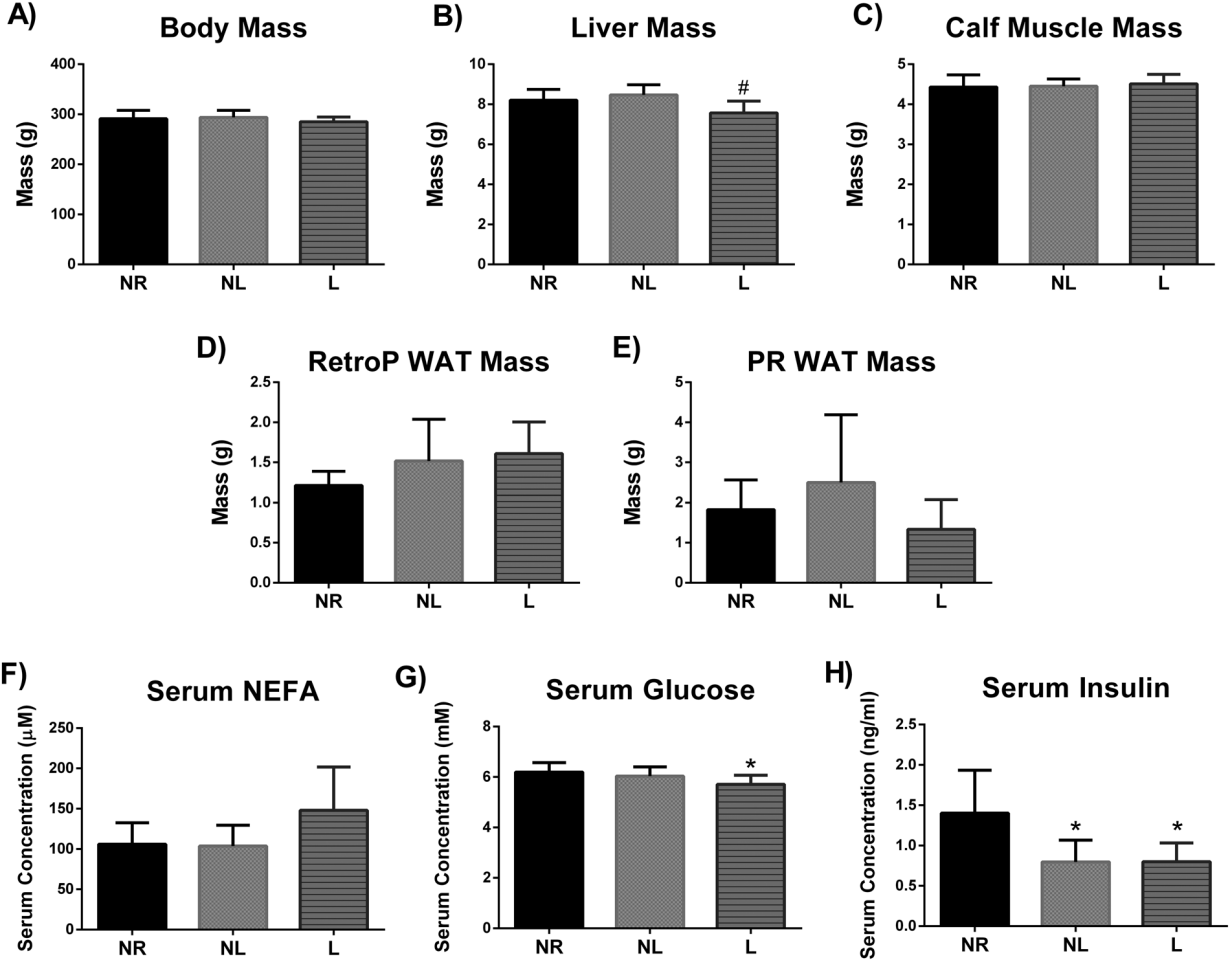
Body mass, tissue mass, and serum metabolites for age-matched rats that did not reproduce (NR), and rats that did not (NL) or did (L) suckle their young. (**A**) Body mass, (**B**) liver mass, (**C**) calf muscle mass of both rear triceps surae, (**D**) retroperitoneal (RetroP) white adipose tissue (WAT) mass, (**E**) perirenal (PR) WAT mass, (**F**) serum concentration of non-esterified fatty acids (NEFA), (**G**) serum concentration of glucose, and F) serum concentration of insulin. Data shown are mean ± SD (n = 7–8 per group). *Different from NR, and # different from NL (p < 0.05).

Lactation has been demonstrated to impact body composition following parturition^13^. Liver mass was lower in L compared to NL (p = 0.017). However, no statistical changes were observed in body mass, combined mass of triceps surae calf muscle, RetroP WAT, or PR WAT following a 12-week period post lactation (p > 0.05) (Fig. 1A–E).

### Serum glucose, insulin, and NEFA concentrations

Dysregulation of two important blood metabolites, glucose and NEFA, can be indicative of metabolic disease. Considering that regulation of glucose and NEFA must occur during lactation, we sought to determine if these effects persist 12-weeks following lactation. No statistical differences were detected in serum NEFA concentrations (p > 0.05) (Fig. 1F). Serum glucose concentrations were lower in L compared to NR (p = 0.046) (Fig. 1G). Additionally, serum insulin concentrations were lower in both NL and L compared to NR (p = 0.007).

### Whole animal metabolism

**Figure 2:**
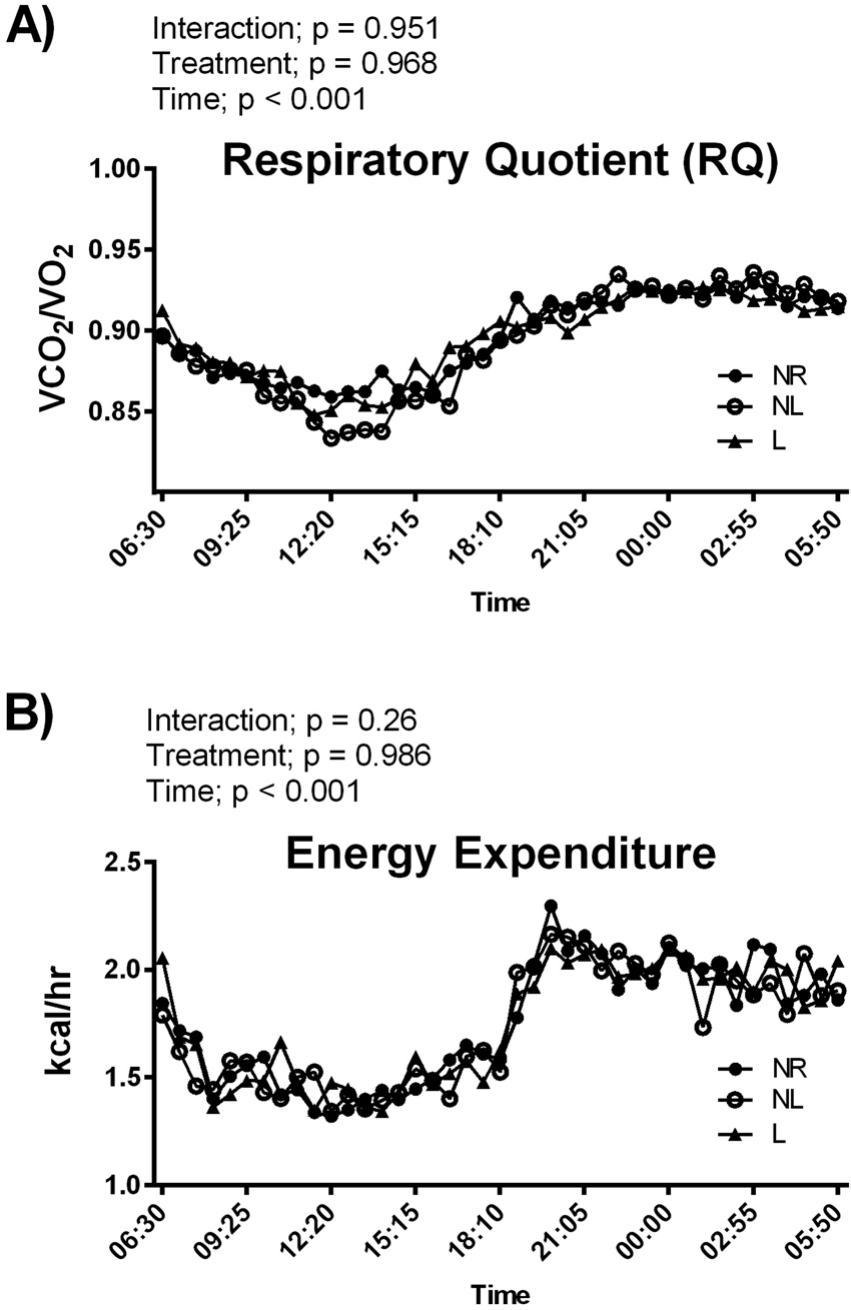
Whole animal metabolism for age-matched rats that did not reproduce (NR), and rats that did not (NL) or did (L) suckle their young for 21 days postpartum (n = 7–8 per group). (**A**) Respiratory quotient and (**B**) energy expenditure.

In order to observe if lactation resulted in changes in whole body metabolism, 24 hour energy expenditure (EE) and respiratory quotient (RQ) were assessed using metabolic chambers. RQ and EE data showed a significant time effect (p < 0.001), but no statistical differences were detected between groups (p > 0.05) (Fig. 2A and B).

### Mitochondrial function and oxidant emission

**Figure 3:**
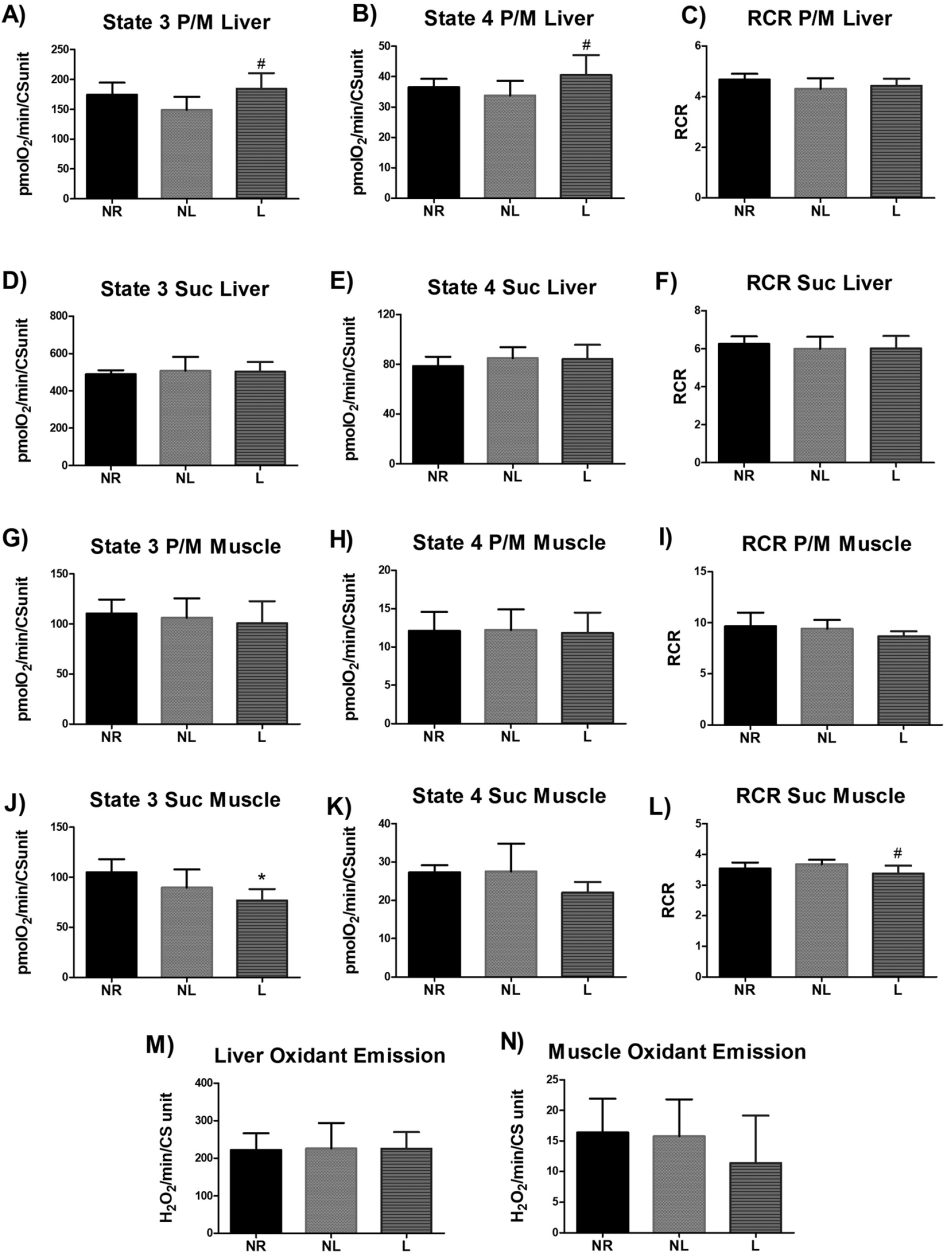
Respiration and oxidant emission from isolated mitochondria for age-matched rats that did not reproduce (NR), and rats that did not (NL) or did (L) suckle their young. Data include liver (**A**) state 3 respiration utilizing complex I substrates (pyruvate and malate; P/M), (**B**) state 4 respiration utilizing P/M, (**C**) respiratory control ratio (RCR) utilizing P/M), (**D**) state 3 respiration utilizing complex II substrates (succinate; suc), (**E**) state 4 respiration utilizing Suc, (**F**) RCR utilizing suc. Data also include skeletal muscle (**G**) state 3 respiration utilizing P/M, (**H**) state 4 respiration utilizing P/M, (**I**) RCR utilizing P/M, (**J**) state 3 respiration utilizing suc, (**K**) state 4 respiration utilizing suc, (**L**) RCR utilizing suc. Finally (**M**) oxidant emission from liver and (**N**) oxidant emission from skeletal muscle are also presented. Oxygen consumption and hydrogen peroxide (H_2_O_2_) rates are normalized to citrate synthase (CS). Data shown are mean ± SD (n = 6–8 per group). *Different from NR, and # different from NL (p < 0.05).

**Figure 4:**
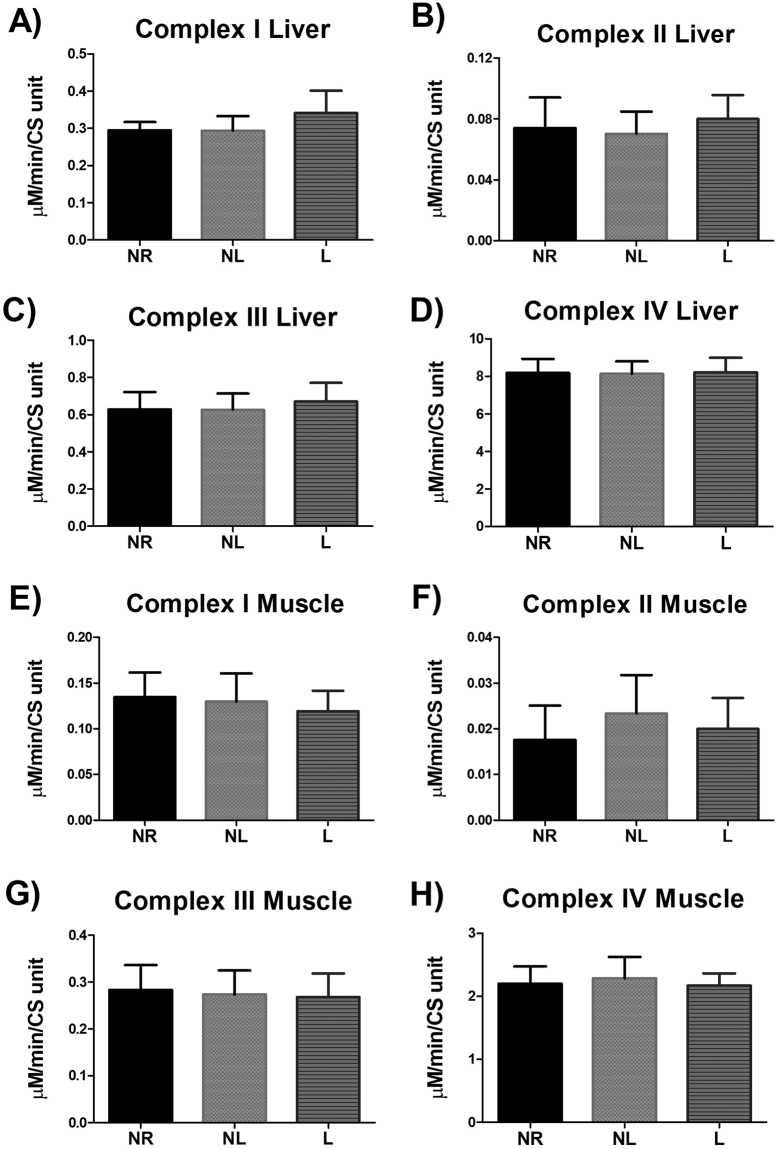
Enzymatic complex activity of isolated mitochondria for age-matched rats that did not reproduce (NR), and rats that did not (NL) or did (L) suckle their young. Data include (**A**) complex I, (**B**) complex II, (**C**) complex III, and (**D**) complex IV activity in the liver. In addition, data include (**E**) complex I, (**F**) complex II, (**G**) complex III, and (**H**) complex IV activity in skeletal muscle. Complex activity data are normalized to citrate synthase (CS). Data shown are mean ± SD (n = 6–8 per group). No statistical differences detected (p > 0.05).

Measurements of isolated mitochondrial respiration rates provide insight to the capacity to utilize substrate and produce ATP. State 3 and state 4 respiration was higher in liver mitochondria in L compared to NL (p = 0.025 and p = 0.041, respectively) when utilizing complex I substrate (pyruvate/malate (P/M)) (Fig. 3A and B). State 3 respiration in skeletal muscle was lower in L compared to NR (p = 0.004) when utilizing the complex II substrate succinate (Fig. 3J), and skeletal muscle RCR was lower in L compared to NL (p = 0.045) (Fig. 3L). No statistical differences were detected in oxidant emission or enzymatic activity of mitochondrial complex activity in liver or skeletal muscle (p > 0.05) (Figs 3 and 4).

### Markers of metabolism

**Figure 5:**
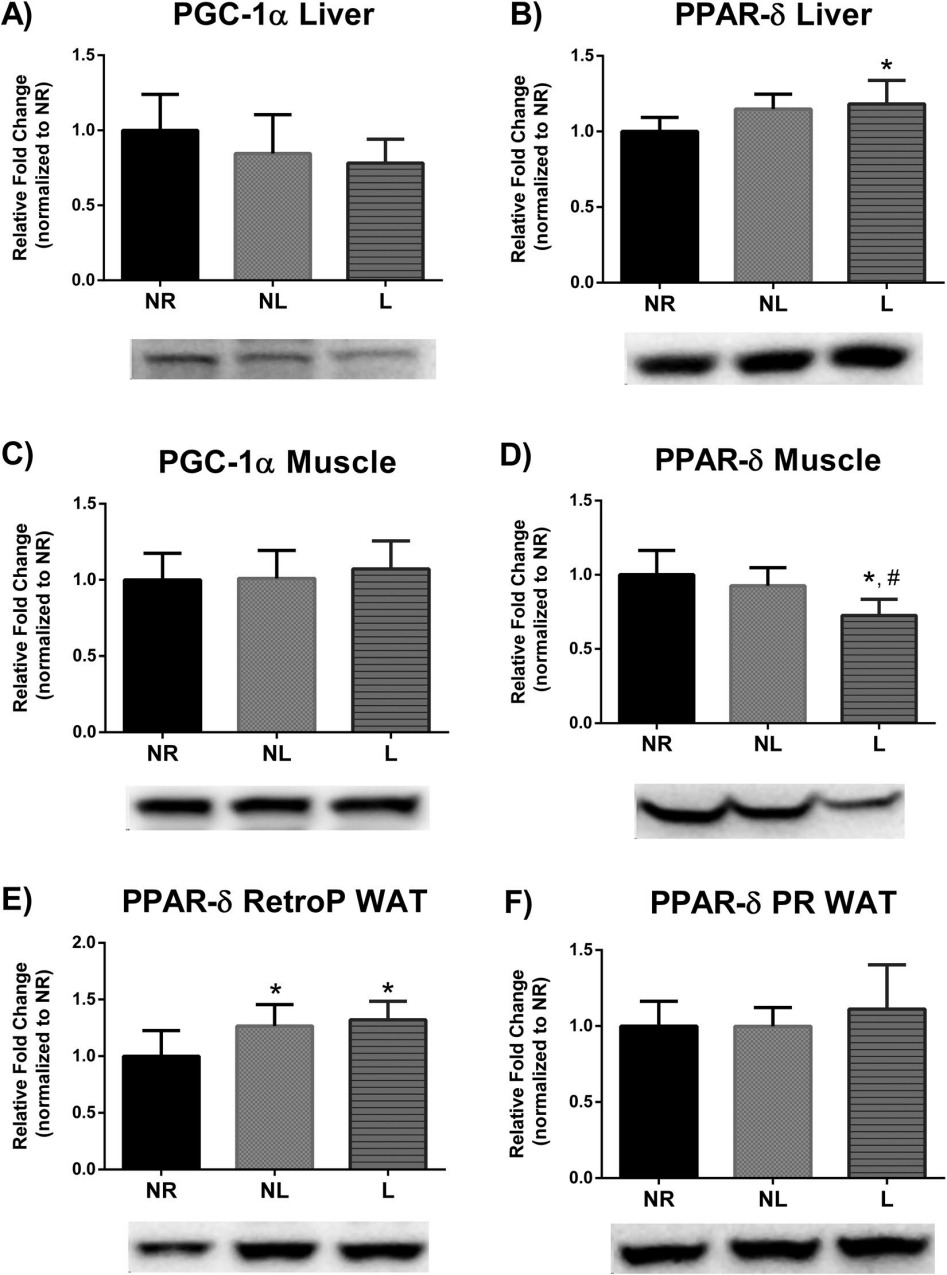
Markers of metabolism in liver, skeletal muscle, and Retroperitneal (RetroP) and perirenal (PR) white adipose tissue (WAT) for age-matched rats that did not reproduce (NR), and rats that did not (NL) or did (L) suckle their young. Data include (**A**) PGC-1α and (**B**) PPARδ protein levels in liver. (**C**) PGC-1α and D) PPARδ protein levels in skeletal muscle. (**E**) PPARδ protein levels in WAT. Representative blots are shown under the graphs. Images are shown as captured, protein expression was normalized to Ponceau staining, and variability between membranes was normalized by using an internal control run with each membrane. Data shown are mean ± SD (n = 6–8 per group). *Different from NR, and ^#^Different from NL (p < 0.05).

The PPAR superfamily is associated with the regulation of genes involved in oxidative metabolism. Specifically, PPARδ is associated with genes involved in lipid and glucose metabolism^29,30^. Liver PPARδ protein levels were higher in L compared to NR (p = 0.024) (Fig. 5B). Skeletal muscle PPARδ protein levels were lower in L compared to NR and NL (p = 0.005 and p = 0.039, respectively) (Fig. 5D). PPARδ protein expression in RetroP WAT was higher in NL and L compared to NR (p = 0.04 and p = 0.009, respectively) (Fig. 5F).

### Markers of oxidative stress

**Figure 6:**
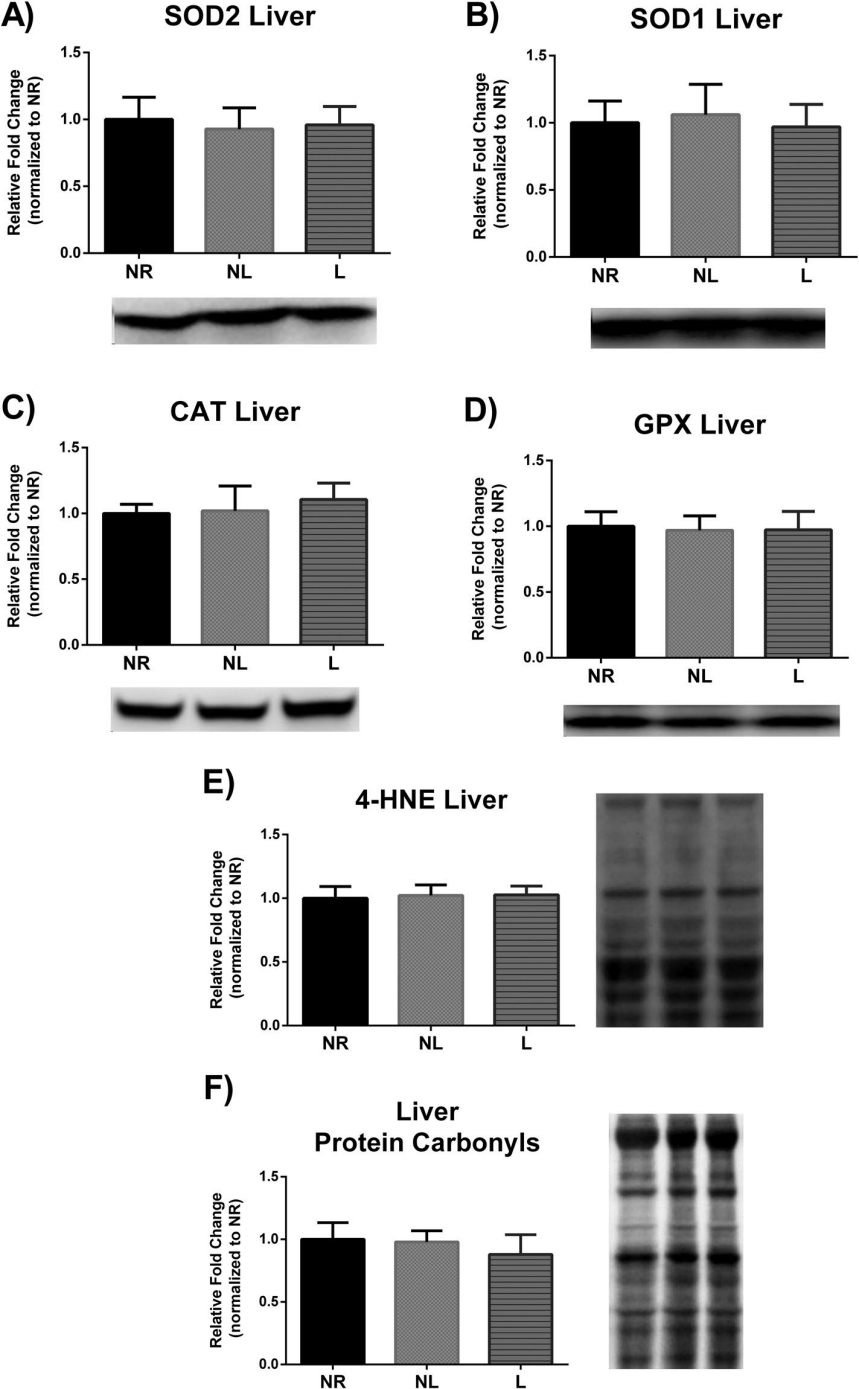
Markers of oxidative stress in liver for age-matched rats that did not reproduce (NR), and rats that did not (NL) or did (L) suckle their young. Data include protein level of the antioxidants (**A**) SOD2, (**B**) SOD1, (**C**) CAT, and (**D**) GPX. In addition, data include markers of oxidative damage including (**E**) lipid peroxidation determined by 4-HNE, and (**F**) protein carbonyls levels determined by using oxyblot. Representative blots are shown under the graphs (**A**–**D**) or to the right of the graphs (**E** and **F**). Images are shown as captured, protein expression was normalized to Ponceau staining, and variability between membranes was normalized by using an internal control run with each membrane. Data shown are mean ± SD (n = 6–8 per group). No significant differences detected (p > 0.05).

**Figure 7:**
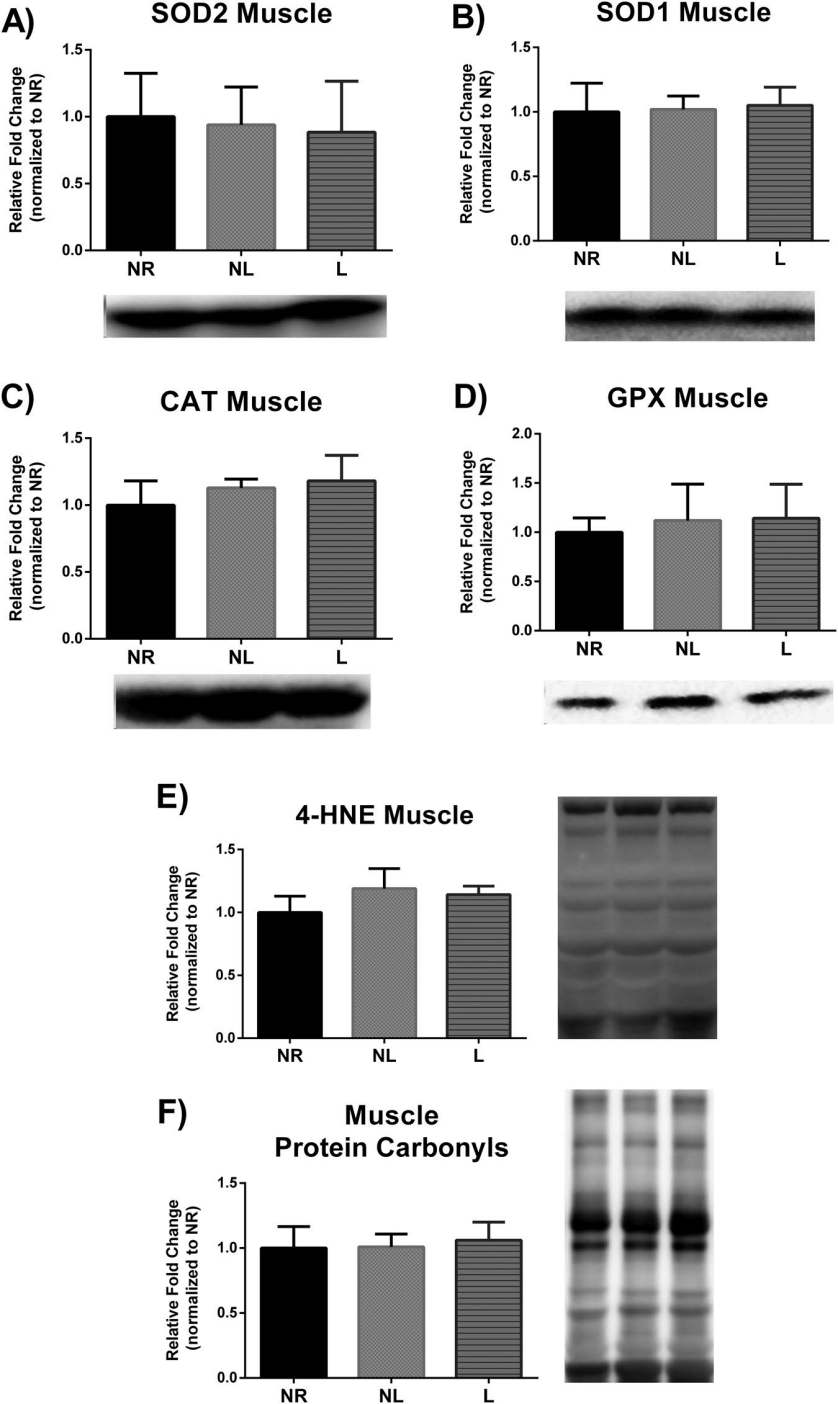
Markers of oxidative stress in skeletal muscle for age-matched rats that did not reproduce (NR), and rats that did not (NL) or did (L) suckle their young. Data include protein level of the antioxidants (**A**) SOD2, (**B**) SOD1, (**C**) CAT, and (**D**) GPX. In addition, data include markers of oxidative damage including (**E**) lipid peroxidation determined by 4-HNE, and (**F**) protein carbonyls levels determined by using oxyblot. Representative blots are shown under the graphs (**A**– **D**) or to the right of the graphs (**E** and **F**). Images are shown as captured, protein expression was normalized to Ponceau staining, and variability between membranes was normalized by using an internal control run with each membrane. Data shown are mean ± SD (n = 5–8 per group). No significant differences detected (p > 0.05).

**Figure 8:**
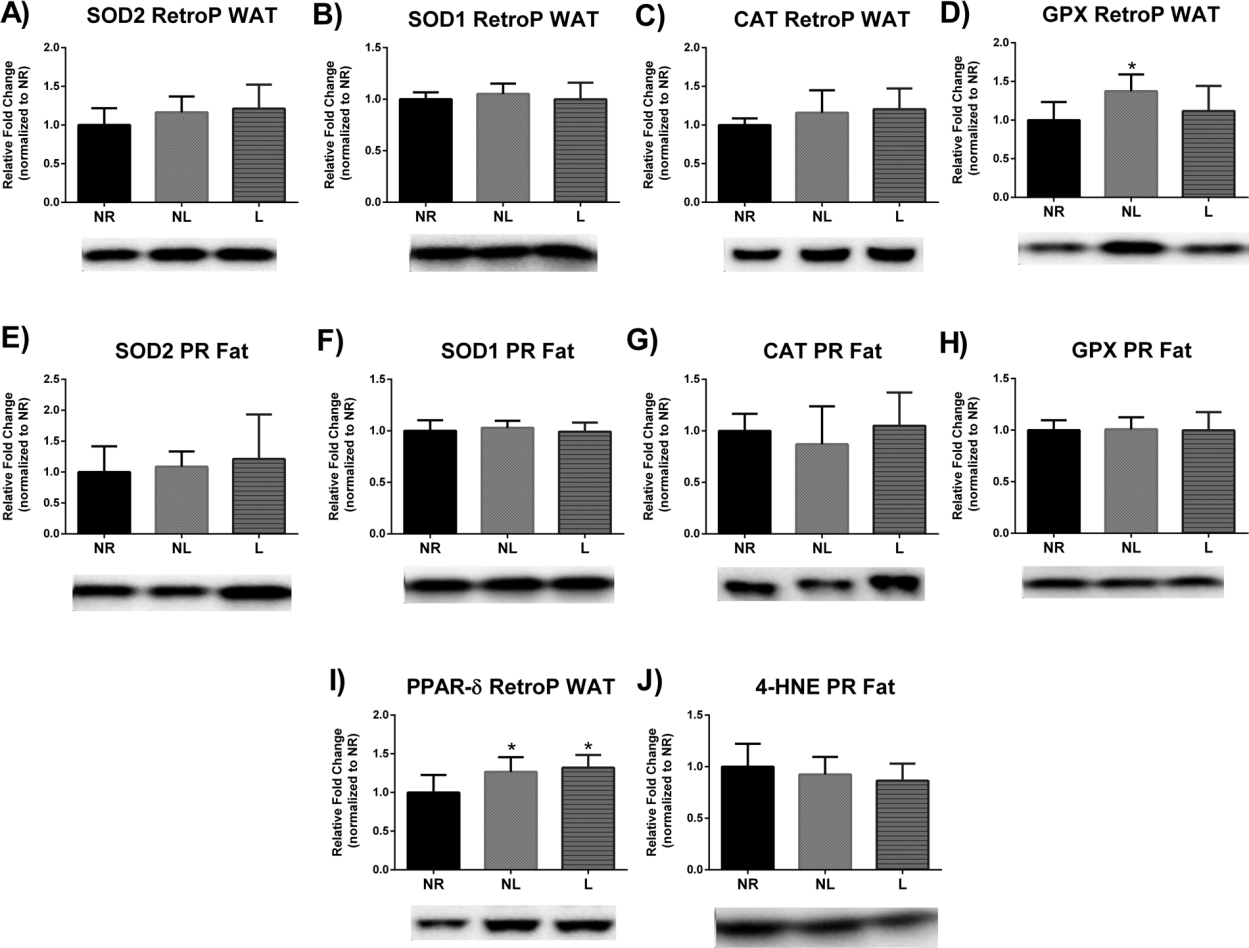
Markers of oxidative stress in retroperitoneal white adipose tissue (RetroP WAT) and perirenal (PR) WAT for age-matched rats that did not reproduce (NR), and rats that did not (NL) or did (L) suckle their young. Data include RetroP WAT and PR WAT protein level of the antioxidants SOD2, SOD1, CAT, and GPX (**A**–**H**). In addition, data include lipid peroxidation determined by 4-HNE in RetroP and PR WAT. Representative blots are shown under the graphs. Images are shown as captured, protein expression was normalized to Ponceau staining, and variability between membranes was normalized by using an internal control run with each membrane. Data shown are mean ± SD (n = 6–8 per group). *Different from NR (p < 0.05).

The balance between oxidants and antioxidants is often referred to as oxidative stress. Endogenous antioxidant defense mechanisms exist to protect and detoxify oxidants. Specifically, SOD2 and SOD1 act to detoxify superoxide into the lesser harmful hydrogen peroxide. Hydrogen peroxide can be then further detoxified by CAT and GPX. No statistical differences were detected in liver (Fig. 6), skeletal muscle (Fig. 7) in any of the aforementioned oxidative stress markers. However, GPX protein levels were higher in RetroP WAT of NL compared to NR (p = 0.040), but no other differences were reported in either RetroP or PR WAT (Fig. 8).

## Discussion

Epidemiological reports show that breastfeeding decreases the prevalence of several maternal health disparities late in life and Stuebe and Rich-Edwards proposed that lactation may reset a female’s probability of metabolic disease^14^. In the current study, we used a rat experimental model to characterize the effects of lactation on a female’s metabolism. We report that at 7 months of age, about 4 months after parturition, lactating females had lower serum glucose concentration and differing PPARδ protein levels in liver, skeletal muscle, and WAT than age match females in the other groups. Furthermore, the capacity of mitochondrial respiration was increased in the liver of animals that lactated, while a decrease was observed in skeletal muscle mitochondrial respiration. Our novel findings suggest that lactation does not merely reset a female’s metabolism but instead contributes to metabolic changes in multiple tissues that could confer greater resistance to metabolic disease than found in both females that gave birth and did not lactate and females that do not reproduce at all. Further discussion of our results follows.

### Lactation has lasting effects on metabolism

We found no differences in body mass or body composition (muscle or WAT mass) between groups. Several reports have shown that prevalence of obesity is decreased later in life in women who breastfed compared to those that did not^1,2,31^. Nevertheless, it is not surprising that we did not find differences in our NR, NL, and R rats given that rats have a dramatically greater mass specific metabolic rate and are less likely to over eat than humans. In addition, rats in this study had not achieved maximum body size at the time of parturition but most published studies evaluating the impact of lactation on maternal phenotype in humans were conducted in countries where women typically reach adult size before pregnancy. These differences between species in relative size during reproduction may have also contributed to why the predicted differences in phenotype were not observed. Nevertheless, rats in the NR, NL, and L groups did display differences in metabolic markers and mitochondrial function at 7 month of age.

Specifically, liver PPARδ protein levels were higher in animals that lactated. Previously, Sanderson and collaborators utilized a knockout model of PPARδ, and observed lower expression of genes relating to glucose metabolism in the liver and higher plasma glucose in a fasted state compared to wild type mice^32^. Similarly, we demonstrate that L rats with higher liver PPARδ protein levels and had lower serum glucose concentrations, suggesting that these rats metabolize glucose more efficiently. It also important to note that both NL and L rats had lower serum insulin levels compared to NR rats, but the serum glucose of NL rats was similar to NR rats. An increase in the capacity of the liver of L rats to metabolize glucose is further supported by our findings of increased state 3 and state 4 respiration in liver mitochondria when a complex I substrate (P/M) was used as the fuel. A higher state 3 mitochondrial respiration indicates a higher maximal capacity for ATP production, but also an increase of state 4 respiration may be indicative of an overall increase in energy utilization. State 4 mitochondrial respiration can be used as a proxy for leak respiration. Leak respiration has been linked to increases in energy expenditure due to its involvement in multiple roles such as thermogenesis and reducing oxidant production in the mitochondria^33^. Thus, our novel findings of increased PPARδ expression in liver, increased mitochondrial respiration in liver, and decreased serum glucose provides evidence of mechanistic events in which lactation may be protective against the development of type II diabetes: supporting the lactation reset hypothesis. However, it is important to note that PPARδ nuclear localization and phosphorylation can affect its activity and thus follow up studies should expand on our findings. Furthermore, liver mass was decreased in animals that lactated. It is feasible that this difference was reflective of decreased hepatic fat content, this would reduce risk of developing type II diabetes, considering hepatic fat content is associated with insulin resistance^34^. Our experimental design did not include measurements of hepatic fat and warrants further research.

We do not report any differences between groups in energy expenditure. However, measurements taken in the metabolic cages reflect whole body metabolism and do not provide information on relative metabolism of different tissue types. Indeed, our results would suggest a shift in tissue metabolism. PPARδ protein levels were lower in skeletal muscle of L rats, which counters our findings for liver. PPARδ regulates the expression of genes involved in lipid metabolism in skeletal muscle^35^, therefore a decrease in PPARδ in skeletal muscle may indicate reduced lipid oxidation. Indeed, rats that lactated had a lower skeletal muscle state 3 mitochondrial respiration when a complex II substrate was utilized. Considering that beta-oxidation results in a higher FADH/NADH ratio, this can be considered a decrease in the capacity to utilize substrate from oxidized lipid sources. A decrease in lipid metabolism in skeletal muscle and an increase in glucose metabolism in liver of rats that lactated would act to balance out our observed similarities of total energy expenditure between groups. Furthermore, PPARδ protein levels in RetroP WAT were higher in rats that reproduced and PPARδ plays a catabolic role in WAT via lipid oxidation^12,30^. Our findings of differing PPARδ protein levels in liver, skeletal muscle, and WAT indicates that a systemic signaling event drives many of the metabolic changes with lactation, however the specific upstream regulator of PPARδ in lactation is currently unknown and warrants further research.

### Oxidative stress

Reactive oxygen species (ROS) are capable of damaging cellular structures; but ROS are a naturally occurring phenomenon that can manifest during energy production in the mitochondria. While ROS can also serve as important signaling stimuli, a balance must be maintained between oxidants and antioxidants to avoid cellular damage; this balance is referred to as oxidative stress. In this regard, the free radical theory of aging posits that ROS production over a lifetime leads to cumulative damage causal in cellular senescence. Furthermore, the disposable soma theory of aging postulates that participation in reproduction detracts from available resources normally utilized for somatic maintenance (e.g. endogenous antioxidant defenses)^36^. Given that a heightened energetic demand is required during reproduction and thus a greater opportunity for ROS production, researchers have posited that reproduction poses a life-tradeoff between longevity and reproduction^37–39^. However, mechanistic evidence supporting increased oxidative stress due to reproduction remains equivocal^40,41^. On the contrary, several reports have actually observed decreased markers of oxidative stress in liver during lactation^42,43^. Indeed, other metabolic challenges such as exercise have actually shown to provide a hormetic response to oxidative stress^2^. Nonetheless, our findings demonstrate no changes between groups in oxidative damage for skeletal muscle, liver, or WAT 4 months after parturition. Thus, it appears unlikely that one bout of reproduction would have a detrimental impact on longevity via oxidative stress and it also appears unlikely that oxidative stress contributes to differences in developing metabolic disease, such as obesity, type II diabetes, and cancer^18,19,44^ in women who do not breastfeed.

## Conclusions

To our knowledge, we are the first to observe the prolonged effects of one bout of reproduction (with or without lactation) on whole animal, molecular, and cellular markers. While we did not observe difference in body mass, skeletal muscle, or WAT between groups, we report that liver mass was lower in animals that lactated 12 weeks following the weaning of pups. We report novel findings of altered markers of metabolism and mitochondrial function in liver and skeletal muscle in animals that lactated. Specifically, serum glucose concentrations were lower in animals that lactated. Importantly, we report altered PPARδ levels in liver, skeletal muscle, and WAT. Furthermore, we observed that mitochondrial function is altered in liver and skeletal muscle 12 weeks following the cessation of lactation, such that respiratory capacity is increased in liver and decreased in skeletal muscle of animals that lactated. Finally, we report that reproduction did not result in any lasting effects in oxidative damage or antioxidant levels. The reset hypothesis of Stuebe and Rich-Edwards^14^ implied that lactation reverses many of the metabolic changes that occur during pregnancy. Our results suggest that lactation goes beyond returning females back to a non-reproductive baseline and improves their metabolic condition long after reproduction has ended. As a limitation to this study, we note that that research has shown that mother-pup separation may have neurobehavioral and physiological effects on both the mother and the pups^45,46^, and future research is needed to expound on our findings and to determine the effects of these differences on health parameters.
